# PLASMA PROTEIN BIOMARKERS AND THEIR CORRELATION TO COGNITIVE PERFORMANCE IN WOMEN LIVING WITH HIV

**DOI:** 10.1093/geroni/igae098.2784

**Published:** 2024-12-31

**Authors:** Wei Li, Ge Wang, David Vance, Daniel Li

**Affiliations:** University of Alabama at Birmingham, Birmingham, Alabama, United States; Tongji Medical College of Huazhong University of Science and Technology, Wuhan, Hubei, China (People’s Republic); University of Alabama at Birmingham, Birmingham, Alabama, United States; UCLA, Los Angeles, California, United States

## Abstract

**Background:**

Individual plasma protein biomarkers have been shown to correlate to cognitive performance in people with HIV (PWH). This study aimed to investigate the association between plasma proteomic signatures and cognition in virologically well-controlled women with HIV (WWH).

**Methods:**

Seventy-seven WWH from three Women’s Interagency HIV Study (WIHS) sites completed neuropsychological (NP) testing and a blood draw. Targeted protein biomarkers were analyzed using a multiplexing method. Random forest analysis was used to identify the top 10 positively or negatively biomarkers associated with cognitive function. Next, ingenuity pathway analysis (IPA) was used to facilitate data interpretation.

**Results:**

Tumor necrosis factor receptor 1 (TNF RI), TNF RII, interleukin 1 receptor 1 (IL-1RI), and IL-6R are negatively associated with attention/working memory; IL-7 and CD40 are positively associated with executive function; IL-1RI, hepatocyte growth factor (HGF), transforming growth factor beta-1 (TGFβ1), TGFβ2, and sonic hedgehog (SHH) are negatively associated but IL-7 is positively associated with fluency; IL-9, IL-15, and IL-21R are positively associated with motor function; brain-derived neurotrophic factor (BDNF), HGF, SHH, and vascular endothelial growth factor (VEGF) are negatively associated with processing speed. HGF, VEGF, and insulin are negatively associated with the global NP function. Based on the IPA, one to three signaling networks were proposed for associating plasma biomarkers with each domain-specific or global NP function.

**Discussion:**

It is likely that a panel of plasma protein biomarkers can be used to predict domain-specific or global NP function. Multiple signaling networks might explain cognitive functioning in WWH.

